# Lipid remodeling and ferroptosis in phosphatidylcholine-mediated tumor–stroma crosstalk

**DOI:** 10.3389/fmolb.2026.1742305

**Published:** 2026-01-28

**Authors:** Manikandan Vani Raju, Meenakshi Kaniyur Chandrasekaran, Lavanya Loganathan, Rathi Muthaiyan Ahalliya, Monica Mironescu, Ion Dan Mironescu, Chella Perumal Palanisamy, Gopalakrishnan Velliyur Kanniappan

**Affiliations:** 1 Department of Biochemistry, Karpagam Academy of Higher Education, Coimbatore, Tamil Nadu, India; 2 Natural Products and Cancer Biology Lab, Department of Physiology, Saveetha Medical College and Hospital, Saveetha Institute of Medical and Technical Sciences (SIMATS), Chennai, Tamil Nadu, India; 3 Department of Biotechnology, Karpagam Academy of Higher Education, Coimbatore, Tamil Nadu, India; 4 Faculty of Agricultural Sciences Food Industry and Environmental Protection, Lucian Blaga University of Sibiu, Sibiu, Romania; 5 Phytochemistry and Pharmacotherapy Lab, Department of Dermatology, Saveetha Medical College & Hospital, Saveetha Institute of Medical and Technical Sciences (SIMATS), Chennai, Tamil Nadu, India

**Keywords:** ferroptosis, lipid peroxidation, phosphatidylcholine, PUFAs, tumor microenvironment

## Abstract

Tumor progression and therapy resistance are formed not only by cancer cell intrinsic mechanisms but also by dynamic interactions with the surrounding tumor stroma. Metabolic regulation is central to these interactions, with lipid pathways emerging as critical determinants of cell fate. A growing concept is the phosphatidylcholine ferroptosis axis, which functions as a metabolic checkpoint linking membrane remodeling, oxidative stress, and tumor stroma communication. Phosphatidylcholine, the most abundant phospholipid in mammalian membranes, is primarily synthesized through the Kennedy pathway under the control of choline kinase alpha. Enhanced phosphatidylcholine synthesis alters membrane architecture and generates substrates for lipid remodeling. These changes regulate susceptibility to ferroptosis, a regulated form of cell death driven by iron-dependent lipid peroxidation. Remodeling by enzymes such as lysophosphatidylcholine acyltransferases and phospholipase A2 influences polyunsaturated fatty acid incorporation into membranes, thereby determining the pool of lipids prone to oxidative damage. Phosphatidylcholine metabolism extends beyond cancer cells to the tumor microenvironment. Cancer-associated fibroblasts can supply phosphatidylcholine derived from metabolites that buffer tumor cells against ferroptosis, while altered phosphatidylcholine dynamics modulate macrophage and T cell activity, influencing immune surveillance and inflammatory tone. Through these mechanisms, the phosphatidylcholine–ferroptosis axis integrates metabolic control of cell death with stromal regulation of tumor growth and resistance. Therapeutically, targeting phosphatidylcholine synthesis and remodeling combined with ferroptosis inducers or glutathione peroxidase 4 inhibitors could sensitize tumors to oxidative cell death while reprogramming stromal elements toward anti-tumor activity. This places the phosphatidylcholine ferroptosis axis as a promising target for combination therapies designed to disrupt tumor stroma crosstalk and improve clinical outcomes.

## 1 Introduction

The tumor microenvironment (TME) is a highly dynamic and complex system composed of both cancerous and non-cancerous cells that collectively influence tumor progression, metastasis, and response to therapy. A key aspect of this environment is the continuous and coordinated crosstalk between tumor cells and stromal elements such as cancer-associated fibroblasts (CAFs), immune cells, endothelial cells, and pericytes. Tumor-stroma interactions transform the TME from a passive space into an active participant in malignancy (Kalluri, 2016). CAFs, which are activated by tumor-derived factors like TGF-β, undergo functional changes that lead them to remodel the extracellular matrix (ECM), producing dense collagen that increases tissue stiffness and promotes cancer cell proliferation through mechanotransduction, while also forming structural pathways for tumor invasion. At the same time, CAFs and cancer cells secrete chemokines that attract immune cells and shift them toward pro-tumoral roles. This includes the polarization of tumor-associated macrophages (TAMs) to an M2 phenotype and the expansion of myeloid-derived suppressor cells (MDSCs), both of which suppress anti-tumor immune responses by depleting essential amino acids and expressing immune checkpoint ligands such as PD-L1, leading to T cell exhaustion and exclusion (Shi Z. et al., 2025). Moreover, the vascular system in the TME becomes abnormal due to sustained signaling by angiogenic factors like VEGF, which drive endothelial cells to form disorganized, leaky, and unstable vessels lacking proper pericyte coverage. These defective blood vessels raise interstitial pressure, hinder drug delivery, and create hypoxic regions that further promote tumor aggression and metastasis (Ha et al., 2025). Endothelial cells and pericytes, influenced by CAF-secreted factors and signals such as TGF-β, actively contribute to this pathological state. For instance, pericytes, traditionally seen as vascular stabilizers, respond to tumor-derived signals and secrete molecules like IGFBP-3 that enhance invasion and chemoresistance (Navarro et al., 2020). Collectively, this triad of ECM remodeling, immune suppression, and dysfunctional vasculature facilitated by a network of signaling molecules and extracellular vesicles creates a self-sustaining ecosystem that supports every hallmark of cancer, including proliferation, survival, invasion, and therapeutic resistance (Quail and Joyce, 2013). Thus, the TME, driven by the active participation of stromal components like CAFs, immune cells, endothelial cells, and pericytes, plays a central role in tumor biology and represents a critical target for future cancer therapies (Räsänen and Vaheri, 2010; Liu et al., 2019; Wang et al., 2020; de Visser and Joyce, 2023; Gao et al., 2023; Xiao et al., 2024).

Bidirectional signaling between malignant cells and surrounding stromal and immune components forms a core mechanism that maintains and advances the TME. This communication occurs through cytokines, chemokines, metabolites, and extracellular vesicles (EVs), especially exosomes, allowing a constant exchange of molecular information that shapes the behavior of multiple cell types (de Goede et al., 2020; Li X. et al., 2022). Malignant cells release factors like TGF-β to transform normal fibroblasts into CAFs, which then secrete signals such as HGF and CXCL12 that further support cancer cell proliferation and invasion, creating a self-sustaining pro-tumorigenic loop (Filippakopoulos et al., 2012). Exosomes act as intercellular carriers, transporting proteins, lipids, and non-coding RNAs that can reprogram recipient cells to promote angiogenesis, immune evasion, or therapy resistance (Yilmaz et al., 2023). A critical aspect of this crosstalk is metabolic, forming a symbiotic relationship where oxidative tumor cells induce nearby CAFs to shift toward glycolysis. These glycolytic CAFs produce energy-rich metabolites like lactate, which are exported and taken up by cancer cells via monocarboxylate transporters to fuel mitochondrial oxidative phosphorylation and tumor expansion (Lee et al., 2016). Stromal cells, including fibroblasts and adipocytes, also contribute fatty acids and amino acids to support the metabolic demands of tumors (Attané and Muller, 2020; Comito et al., 2020). Simultaneously, metabolic antagonism allows tumor cells to suppress immune function by depleting essential nutrients like glucose and glutamine, limiting immune cell activity (Basil and Levy, 2015; Chen et al., 2022; Yang et al., 2025). High lactate levels from tumor metabolism also acidify the TME, directly impairing the function of cytotoxic T lymphocytes and NK cells (Belkahla et al., 2022). Additionally, tumor cells upregulate enzymes like indoleamine 2,3-dioxygenase, leading to tryptophan breakdown into kynurenine, which causes T-cell apoptosis and supports regulatory T-cell formation (Zhou et al., 2024). Through this combination of molecular signaling, exosome transfer, and metabolic rewiring, tumor cells actively reprogram their microenvironment to sustain cancer hallmarks, suppress immunity, and resist therapy (Turdo et al., 2020). This complex metabolic and immunological network positions the TME as a dynamic, adaptive, and drug-resistant ecosystem central to cancer progression.

Metabolic checkpoints are key regulatory nodes within the cellular bioenergetic network that sense nutrient availability and metabolic flux to determine cell fate decisions, such as whether a cell will proliferate, remain quiescent, differentiate, or undergo apoptosis. Much like immune checkpoints that regulate T-cell responses, metabolic checkpoints act as molecular sensors that integrate signals from glucose, amino acids, fatty acids, oxygen, and metabolites like NAD^+^ and acetyl-CoA to guide essential cellular behaviors. Central to this system are pathways such as mTORC1, which promotes cell growth and anabolic processes when nutrients and growth signals are abundant, and AMPK, which counters mTORC1 activity under energy stress by initiating catabolic programs to restore balance (Samuel and Shulman, 2012). Additionally, HIF-1α acts as a hypoxia-responsive checkpoint, promoting survival by shifting metabolism toward anaerobic glycolysis and supporting angiogenesis in low-oxygen environments (Bakleh and Al Haj Zen, 2025; Bharathy et al., 2025). In the TME, these checkpoints become highly relevant due to the competitive and resource-limited conditions. Cancer cells often acquire mutations such as constitutive activation of the PI3K/AKT pathway that allow them to bypass normal checkpoint controls, maintaining mTORC1 activation even under nutrient-deprived conditions to sustain uncontrolled growth. In contrast, tumor-infiltrating lymphocytes (TILs) face a metabolically suppressive environment marked by glucose and amino acid depletion and elevated levels of immunosuppressive metabolites like lactate. This leads to metabolic checkpoint failure in TILs, where insufficient energy and nutrients prevent effective mTORC1 signaling, resulting in immune cell exhaustion or anergy instead of activation (Basil and Levy, 2015). Thus, mapping the molecular architecture of metabolic checkpoints provides critical insights into cancer biology and immune dysfunction. It also opens avenues for therapeutic strategies that either disrupt tumor-specific metabolic pathways or restore metabolic fitness in immune cells to enhance anti-tumor immunity.

Beyond serving as structural components of cellular membranes and reservoirs for energy storage, lipids and their derivatives act as powerful signaling molecules that play critical roles in regulating the TME. Once considered mere metabolic substrates, lipids are now recognized as second messengers, receptor ligands, and enzymatic cofactors involved in essential cellular processes such as proliferation, apoptosis, inflammation, and motility (Snaebjornsson et al., 2020). In cancer, both malignant cells and associated stromal components display profoundly altered lipid metabolism, producing bioactive lipids that actively promote tumor progression. One key example is prostaglandin E2 (PGE2), an eicosanoid derived from arachidonic acid via cyclooxygenase (COX) enzymes, which is abundantly secreted in the TME. PGE2 facilitates angiogenesis, enhances tumor invasiveness, and suppresses anti-tumor immunity by impairing T-cell function and driving macrophage polarization toward the pro-tumorigenic M2 phenotype (Santiso et al., 2024). Another important signaling axis involves the balance between pro-apoptotic ceramide and pro-survival sphingosine-1-phosphate. Tumors exploit the S1P/S1PR pathway to promote cell survival, proliferation, and immune modulation, including the manipulation of lymphocyte trafficking (Drost and Clevers, 2018). Even lipid droplets once thought to be inert are now known to function as dynamic signaling platforms, regulating fatty acid storage, preventing lipotoxicity, and generating lipid-derived signals. Moreover, cancer cells can reprogram nearby adipocytes to release fatty acids, which they then absorb to support growth and survival. This lipid trafficking extends to immune suppression, as lipid accumulation in tumor-infiltrating lymphocytes leads to metabolic paralysis and exhaustion. Lipid mediators released by adipocytes and tumor-associated macrophages can further activate oncogenic pathways such as PI3K/AKT and NF-κB, fueling tumor growth and immune evasion (Attané and Muller, 2020). Additionally, exosomes enriched lipids can deliver these bioactive molecules to other cells within the TME, altering their metabolism and reinforcing tumor-stroma crosstalk (Li X. et al., 2022). This evolving understanding of lipids as central regulatory agents highlights their significance in shaping tumor behavior and opens new therapeutic avenues aimed at targeting lipid signaling to disrupt tumor progression and immune suppression.

## 2 Phosphatidylcholine-ferroptosis axis

**FIGURE 1:**
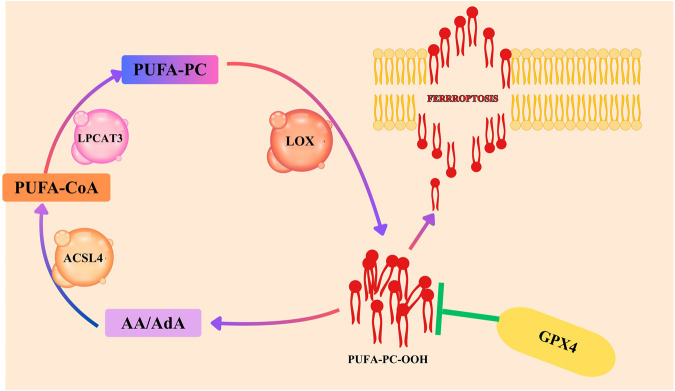
Schematic representation of the Phosphatidylcholine–Ferroptosis axis. Free polyunsaturated fatty acids (PUFAs; arachidonic acid [AA] and adrenic acid [AdA]) are first activated by acyl-CoA synthetase long-chain family member 4 (ACSL4) and subsequently esterified into phosphatidylcholine (PC) through the action of lysophosphatidylcholine acyltransferase 3 (LPCAT3). The resulting PUFA-containing phospholipids are substrates for lipoxygenases (LOX), which catalyze their peroxidation into lipid hydroperoxides (PUFA-PC OOH). Glutathione peroxidase 4 (GPX4) counteracts this process by reducing lipid hydroperoxides to lipid alcohols, thereby suppressing ferroptotic cell death.

Lipid peroxidation is a crucial feature and critical driver of ferroptosis, a regulated form of iron-dependent cell death distinct from apoptosis or necrosis. In ferroptosis, polyunsaturated fatty acids (PUFAs) within membrane phospholipids undergo peroxidation, causing oxidative damage that compromises membrane integrity and leads to cell death. This process is normally prevented by glutathione peroxidase 4 (GPX4), an enzyme that uses glutathione to detoxify lipid hydroperoxides. However, when GPX4 is inhibited or glutathione levels are depleted, lipid peroxides accumulate unchecked, initiating ferroptosis (Kalluri, 2016; Alshammari et al., 2025). The susceptibility of tumor cells to ferroptosis is closely tied to their lipid composition, redox status, and iron availability, positioning lipid peroxidation as both a biomarker and a therapeutic target in cancer. Among phospholipids, only those containing PUFAs such as arachidonic acid (AA) and adrenic acid (AdA) are highly prone to peroxidation due to the chemical reactivity of their bis-allylic hydrogen atoms, which readily undergo hydrogen abstraction and propagate damaging lipid radicals (Samovich et al., 2024). This vulnerability forms the basis of the Phosphatidylcholine–Ferroptosis Axis, a key regulatory pathway linking lipid metabolism to ferroptotic cell death. Phosphatidylcholine (PC), the most abundant phospholipid in mammalian membranes, acts as the structural backbone for fatty acid incorporation. The risk of ferroptosis is not determined by total PC content, but rather by the presence of PC molecules esterified with PUFAs (PC-PUFAs). The enzyme lysophosphatidylcholine acyltransferase 3 (LPCAT3) plays a central role in this axis by catalyzing the incorporation of AA and AdA into lysophosphatidylcholine, generating pro-ferroptotic PC-PUFAs. As illustrated in Figure 1, these PC-PUFAs can then undergo peroxidation by lipoxygenases (LOX), producing lipid hydroperoxides (PC-PUFA-OOH) that drive ferroptotic cell death unless detoxified by GPX4 (Reed et al., 2022). This positions LPCAT3 and related lipid-remodeling enzymes as gatekeepers of ferroptosis sensitivity. Dysregulation of this axis either through enrichment of PUFA-containing phospholipids or depletion of protective monounsaturated fatty acids can tip the balance toward ferroptosis, exposing a metabolic vulnerability that may be therapeutically exploited in cancer cells dependent on specific lipid pathways for survival (Rodencal and Dixon, 2023).

PC, the most abundant phospholipid in eukaryotic cell membranes, plays a crucial role not only in maintaining membrane structure but also in regulating ferroptosis through its dynamic remodeling. The susceptibility of a cell to ferroptosis is closely linked to the composition of its membrane lipids, particularly the presence of PC species enriched with PUFAs like AA and AdA. These PUFA-containing PC molecules serve as primary substrates for lipid peroxidation, making them central drivers of the oxidative membrane damage that initiates ferroptotic cell death (Dixon et al., 2012). The fatty acid profile of PC is not static but is regulated by a continuous lipid remodeling process known as the Lands cycle, which enables selective incorporation of PUFAs into membrane phospholipids. This remodeling determines the pool of oxidizable lipids available in the membrane, and thus, the cell ferroptosis sensitivity. The remodeling process is initiated by acyl-CoA synthetases, such as ACSL4, which activate PUFAs into their acyl-CoA forms. These activated fatty acids are then incorporated into lysophosphatidylcholine by LPCAT3, generating PUFA-enriched PC species highly prone to peroxidation. Together, ACSL4 and LPCAT3 form a critical regulatory axis that determines ferroptotic vulnerability by controlling the abundance of PC-PUFA species within the membrane. This enzymatic machinery effectively acts as a molecular gateway, feeding substrates into the ferroptotic pathway and shaping the membrane’s readiness for iron-catalyzed lipid peroxidation. Therefore, the PC remodeling axis operates through the coordinated activity of ACSL4 and LPCAT3, which dynamically adjusts the incorporation of polyunsaturated fatty acids into membrane phosphatidylcholine. This balance effectively acts as a metabolic control switch, fine-tuning cellular susceptibility to ferroptosis and representing a potential target for cancer therapies that target lipid metabolism vulnerabilities (Li et al., 2024).

The Phosphatidylcholine-Ferroptosis axis operates at a key intersection where intracellular redox status is converted into signaling events that control membrane stability and influence paracrine communication within the TME. Central to this process is lipid peroxidation, which acts not only as a destructive force but also as a form of redox signaling. When lipid hydroperoxides and their electrophilic derivatives are generated, they can activate stress-response pathways if properly controlled. However, if the antioxidant defense systems such as GPX4 or ferroptosis suppressor protein 1 (FSP1) fail to neutralize this oxidative stress, the signal intensifies beyond repairable levels (Bersuker et al., 2019). The result is ferroptosis, marked by the irreversible breakdown of membrane integrity and physical rupture of the plasma membrane, ultimately leading to cell lysis. This terminal event transforms what began as an intracellular metabolic failure into a highly active paracrine signaling cascade. During ferroptotic cell death, the damaged cell releases a mixture of intracellular contents, including damage-associated molecular patterns (DAMPs) such as ATP and HMGB1, as well as oxidized lipid molecules (Liu et al., 2022b). These factors influence nearby cells within the TME including cancer cells, stromal fibroblasts, and immune cells by triggering diverse responses. Depending on the immune context, these signals can either promote an immunogenic response by recruiting phagocytes and enhancing antigen presentation or contribute to a pro-tumorigenic, chronic inflammatory state that suppresses effective immunity (Greten and Grivennikov, 2019). Therefore, the ferroptotic process extends beyond a single cell demise, acting as a broadcasting mechanism that translates metabolic stress into extracellular cues capable of reshaping the cellular and immunological landscape of the TME. Although the correlation between phosphatidylcholine remodeling and ferroptosis sensitivity is well recognized, whether the PC axis functions as an active mechanistic driver or merely reflects downstream metabolic remodeling remains under active investigation. Current evidence indicates that this pathway serves as both a causal regulator and a metabolic indicator of ferroptotic susceptibility. Mechanistically, the ACSL4–LPCAT3 enzymes directly directs ferroptosis execution by generating oxidizable PUFA–PC substrates that act as the molecular fuel for lipid peroxidation. Inhibition or genetic loss of these enzymes markedly reduces ferroptotic cell death, highlighting their rate-limiting and causative role in initiating lipid peroxidation (Merkel et al., 2023). Conversely, changes in PC composition and oxidation status often reflect broader alterations in redox balance and lipid metabolism, suggesting that the PC axis also functions as a biomarker of the cellular oxidative state. Collectively, the PC–ferroptosis axis represents a bidirectional regulatory network in which PC remodeling both drives and reflects ferroptotic potential, thereby linking lipid metabolism, redox regulation, and cell fate determination in cancer (Liang et al., 2022).

## 3 Phosphatidylcholine metabolism as a central modulator of tumor-stroma dynamics

### 3.1 De novo synthesis (Kennedy pathway)

**FIGURE 2:**
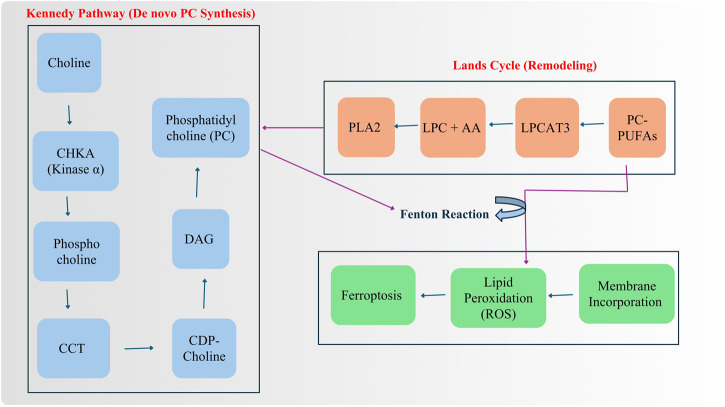
Schematic representation of phosphatidylcholine (PC) metabolism via the Kennedy pathway and Lands cycle. The Kennedy pathway illustrates de novo PC synthesis starting from choline, which is phosphorylated by choline kinase α (CHKA) to form phosphocholine. Phosphocholine is then converted to CDP-choline by CCT, and subsequently combined with diacylglycerol (DAG) to generate phosphocholine (PC). The Lands cycle depicts PC remodeling, in which polyunsaturated PC species (PC-PUFAs) are acylated by LPCAT3 to form lysophosphatidylcholine (LPC) + arachidonic acid (AA), which can be hydrolyzed by PLA2. The remodeled PC species can be reincorporated into membranes or subjected to oxidative stress via the Fenton reaction, leading to lipid peroxidation (ROS) and ferroptotic cell death. Arrows indicate metabolic flux and functional outcomes of PC remodeling.

The Kennedy pathway, which administers the *de novo* synthesis of PC, plays a central role not only in membrane biosynthesis but also in regulating tumor-stroma crosstalk within the TME. This anabolic pathway begins with the uptake of extracellular choline, which is phosphorylated by choline kinase alpha (CHKA) to generate phosphocholine a step frequently upregulated in various cancers including breast, prostate, lung, and colorectal malignancies (Figure 2). Phosphocholine is subsequently converted to CDP-choline by CTP phosphocholine cytidylyltransferase (CCT) and finally condensed with diacylglycerol (DAG) to form PC (Li et al., 2025). This metabolic pathway is often hijacked by oncogenic drivers such as RAS and MYC, which enhance CHKA expression to meet the demands of rapid proliferation and membrane biogenesis (Kortlever et al., 2017). Elevated CHKA activity leads to an accumulation of phosphocholine a recognized metabolic hallmark of malignancy detectable by magnetic resonance spectroscopy (MRS) as part of the total choline signal (Trousil et al., 2014). Clinically, CHKA overexpression correlates with increased tumor grade, aggressiveness, invasion, and poor prognosis. Mechanistically, CHKA also contributes to tumor progression by activating mitogenic signaling pathways such as MAPK and PI3K/AKT, and by interacting with growth factor receptors like EGFR and HER2, indicating non-canonical signaling roles beyond its enzymatic function (Lin et al., 2017). Within the TME, high CHKA expression in tumor cells transforms them into metabolic choline sinks, depriving nearby immune cells, such as tumor-infiltrating lymphocytes (TILs), of choline needed for their own membrane synthesis, thereby contributing to immunosuppression (Li et al., 2025). Additionally, the Kennedy pathway regulates the availability of PC for downstream remodeling into PUFA-containing PC species (PC-PUFAs), which are essential substrates for ferroptotic cell death, and into signaling lipids that facilitate paracrine interactions with fibroblasts, immune cells, and endothelial cells. Thus, this pathway operates at the interface of metabolism and communication, coupling oncogenic lipid synthesis with stromal remodeling and immune evasion, and establishing CHKA as both a functional biomarker and a promising therapeutic target in metabolically reprogrammed cancers.

### 3.2 Remodeling and degradation

PC is not a static membrane component but undergoes continuous remodeling and degradation, processes that critically shape tumor cell behavior and sensitivity to ferroptosis. This remodeling is primarily driven by the Lands cycle, a dynamic process involving deacylation and reacylation of phospholipids. It begins with phospholipase A2 (PLA2) enzymes hydrolyzing the sn-2 fatty acid from PC to generate lysophosphatidylcholine (LPC) and a free fatty acid, which can be utilized for energy or converted into bioactive lipid mediators (Khan and Ilies, 2023). The reacylation step is catalyzed by lysophosphatidylcholine acyltransferases (LPCATs), which reincorporate fatty acyl chains into LPC to regenerate PC (Figure 2). Among these, LPCAT3 plays a key role by preferentially incorporating PUFAs such as AA and AdA, thereby enriching membranes with PC-PUFA species that are highly susceptible to lipid peroxidation (Khan and Ilies, 2023). This activity establishes LPCAT3 as a metabolic gatekeeper that modulates a cell ferroptosis sensitivity, particularly under conditions of oxidative stress common in the TME. A high LPCAT3-mediated flux into PC-PUFA production effectively primes cells for ferroptosis by ensuring an abundant supply of lipid substrates required for iron-dependent oxidative membrane damage. Thus, the interplay between PC synthesis, degradation by PLA2, and selective remodeling by LPCAT enzymes represents a metabolic checkpoint that links membrane lipid composition to redox balance and cell fate decisions. Disruption or manipulation of this remodeling axis offers a promising strategy for therapeutic intervention in cancers dependent on lipid metabolism for survival (Li et al., 2024).

PLA2 enzymes play a central role in the degradation and remodeling of PC by hydrolyzing the sn-2 acyl chain, thereby releasing AA and LPC two bioactive molecules with distinct and critical functions. The sn-2 position of PC is typically enriched with PUFAs, particularly AA, making this cleavage site especially important in cell signaling and inflammation (Lin et al., 2024). Once liberated, AA acts as the primary substrate for the COX and lipoxygenase (LOX) pathways, leading to the synthesis of eicosanoids such as PGE2 and leukotrienes. These lipid mediators are powerful regulators of the TME, promoting angiogenesis, immune suppression, tumor cell invasion, and chronic inflammation (Johnson et al., 2020). Simultaneously, LPC far from being a passive byproduct serves as a key signaling molecule that modulates endothelial permeability, immune cell recruitment, and fibroblast activation. Within the Lands cycle, LPC is also the essential substrate for reacylation by lysophosphatidylcholine acyltransferase (LPCAT) enzymes, especially LPCAT3, which reintroduce specific fatty acids such as AA to regenerate PC-PUFA species that command ferroptosis susceptibility. Thus, PLA2 activity represents a critical metabolic junction that coordinates inflammation, stromal remodeling, and cell death pathways, generating both the signaling lipids that modulate the TME and the oxidizable lipid substrates required for ferroptotic cell death.

Autotaxin (ATX), also known as ectonucleotide pyrophosphatase/phosphodiesterase 2 (ENPP2), is a secreted lysophospholipase D that hydrolyzes LPC into lysophosphatidic acid (LPA) a potent bioactive lipid mediator with wide-ranging effects on the TME. LPC, generated through PLA2-mediated hydrolysis of PC or released from damaged cells, serves as the substrate for ATX, which is often overexpressed and secreted by both tumor cells and CAFs (Laface et al., 2024). The resulting LPA engages a family of G protein-coupled receptors (LPAR1–6) on cancer and stromal cells, triggering downstream pathways such as PI3K/AKT, Rho/ROCK, and MAPK. These cascades collectively drive tumor cell proliferation, survival, invasion, angiogenesis, and immune evasion (van der Ende et al., 2018). Elevated ATX expression and LPA levels are hallmark features of various malignancies and are closely associated with fibrosis, therapeutic resistance, and immunosuppression in the TME. The LPC → LPA axis, composed by ATX, thus serves as a critical extracellular metabolic checkpoint that links lipid turnover to paracrine signaling, transforming a byproduct of membrane degradation into a driver of tumor-stroma crosstalk and making ATX a compelling therapeutic target in cancer (Lancho and Herranz, 2018).

### 3.3 Influence of PC on tumor behavior

PC, beyond its structural role as the most abundant membrane phospholipid, exerts profound regulatory control over tumor cell physiology. The composition and remodeling of PC particularly the acyl chain diversity shaped by the Lands cycle govern membrane fluidity, a key determinant of receptor localization, clustering, and signal transduction. This dynamic property influences critical processes such as growth factor signaling, cytoskeletal remodeling, and cell motility, thereby facilitating invasion and metastasis (Saito et al., 2022). Internally, PC metabolism is tightly linked to endoplasmic reticulum (ER) homeostasis, where imbalances in PC biosynthesis or composition can trigger ER stress and activate the unfolded protein response (UPR). While the UPR initially aims to restore cellular equilibrium, chronic activation in cancer cells often promotes adaptation, survival, and therapy resistance (Sun et al., 2017). Furthermore, PC is essential for autophagy, as the rapid formation of autophagosomal membranes requires a lipid reservoir rich in PC, allowing cancer cells to withstand metabolic stress and nutrient deprivation. PC metabolism also plays a central role in vesicle trafficking, particularly the biogenesis and cargo sorting of exosomes, which are critical for tumor-stroma communication. The lipid composition of these extracellular vesicles affects their stability, targeting, and biological activity. Through exosomal signaling, tumor cells can modulate the behavior of immune cells and fibroblasts, promote angiogenesis, and establish pre-metastatic niches (Ma et al., 2024). Collectively, PC metabolism integrates structural, metabolic, and signaling functions, composing a wide array of tumor-promoting processes and adaptive responses within the TME.

PC metabolism generates potent bioactive lipid messengers most notably lysophosphatidic acid (LPA) and platelet-activating factor (PAF) that coordinate key aspects of tumor progression and intercellular communication within the TME. LPA arises from the extracellular hydrolysis of LPC, itself a product of PLA2-mediated PC cleavage, via the action of autotaxin (ATX/ENPP2). Once generated, LPA binds to a family of G-protein coupled receptors (LPA_1_–_6_) to activate downstream signaling pathways, including PI3K/AKT, Rho/ROCK, and MAPK, thereby enhancing proliferation, migration, angiogenesis, and immune evasion across various cancers (Valdés-Rives and González-Arenas, 2017). Additionally, LPA impairs type I interferon responses in dendritic cells, contributing to immunotherapy resistance (Chae et al., 2022). Parallel to this, PAF is synthesized from LPC through the activity of LPCATs and further remodeled into its bioactive ether form. Acting through the PAF receptor (PAFR) expressed on tumor cells, endothelial cells, and immune populations PAF induces inflammatory signaling, promotes vascular permeability, and facilitates tumor survival and metastasis, particularly under conditions of chemo or radiotherapy-induced stress (Chammas et al., 2017; Ichim and Tait, 2017). Collectively, these PC-derived lipid mediators exemplify how membrane lipid turnover can be repurposed into a highly coordinated signaling platform that sustains malignancy and remodels the TME, marking this axis as a promising target for metabolism-based cancer therapies (Snaebjornsson et al., 2020).

### 3.4 PC metabolism in stromal cells

CAFs exhibit a profoundly reprogrammed lipid metabolism, particularly in PC dynamics, which plays a pivotal role in shaping TME. Compared to normal fibroblasts, CAFs display distinct lipidomic profiles characterized by the accumulation of bioactive lipid species, including LPC and polyunsaturated PC variants prone to peroxidation (Shen et al., 2021). These changes are not passive but are actively involved in metabolic crosstalk with cancer cells. CAFs can export PC, LPC, and fatty acids via exosomes, thereby delivering essential membrane lipids and metabolic intermediates directly to tumor cells. Once taken up, these lipids support membrane biogenesis, redox homeostasis, and serve as substrates for pro-tumorigenic pathways such as the autotaxin–LPA axis (Laface et al., 2024). This intercellular lipid trafficking facilitates nutrient sharing, reinforces immune modulation, and may even influence ferroptosis sensitivity by enriching tumor cell membranes with peroxidation-prone lipids (Morgan et al., 2024). Thus, PC metabolism in CAFs emerges as a non–cell-autonomous regulator of tumor progression, positioning stromal lipid remodeling as a critical determinant of the metabolic and signaling landscape within the TME.

PC metabolism plays a pivotal role in shaping immune cell behavior within the TME, where functional plasticity is tightly coupled to lipid metabolic reprogramming. In macrophages, PC utilization diverges markedly between phenotypes. Pro-inflammatory M1 macrophages, activated by stimuli such as IFN-γ, upregulate *de novo* PC synthesis via the Kennedy pathway to support membrane expansion, phagolysosomal formation, and the production of inflammatory mediators, including eicosanoids derived from arachidonic acid-rich PC species (Wu et al., 2024). This heightened phospholipid flux sustains cytokine secretion and ROS generation, reinforcing anti-tumor activity. In contrast, immunosuppressive M2 macrophages rely on oxidative phosphorylation and fatty acid oxidation, exhibiting altered PC remodeling and reduced phospholipid turnover that favor tissue repair and tumor tolerance. These metabolic profiles are not merely consequences of activation but actively shape bioactive lipid signaling, including the differential release of platelet-activating factors (PAF) and LPC, which further modulate immune-stromal interactions.

A similar dependency on PC metabolism governs T-cell function. Upon activation, naïve T cells undergo a dramatic proliferative burst, necessitating extensive membrane biogenesis. This transition is supported by robust upregulation of PC synthesis, making choline availability within the TME a metabolic checkpoint for effective T-cell expansion and effector function (Okkenhaug, 2013). However, tumor cells through elevated choline uptake and CHKA activity can outcompete T cells for this essential nutrient, inducing a form of metabolic immunosuppression that compromises T-cell activation and persistence (Krug et al., 2024). Thus, PC metabolism acts as a critical regulatory axis for immune cell polarization, effector function, and survival, with tumors exploiting this dependency to create a permissive and immunosuppressive function.

Endothelial cell function is intricately regulated by the acyl chain composition of PC, which modulates membrane biophysics and, consequently, the spatial organization and activation of key signaling proteins. The relative abundance of saturated, monounsaturated (MUFA), and polyunsaturated (PUFA) fatty acids within membrane PC determines fluidity, lipid raft integrity, and receptor clustering, all of which are critical for vascular tone and nitric oxide (NO) signaling (Yaghmour et al., 2025). Specifically, membrane environments enriched in unsaturated PC species particularly MUFAs like oleic acid facilitate the correct localization and activation of endothelial nitric oxide synthase (eNOS) within caveolae, specialized lipid microdomains. This spatial compartmentalization is essential for eNOS to generate NO, a master regulator of vasodilation, inflammation, and vascular barrier function. In contrast, PC species enriched in saturated fatty acids disrupt membrane architecture, destabilize caveolae, and impair eNOS function, promoting its uncoupling into a dysfunctional state that produces superoxide radicals instead of NO (Esposito et al., 2024). Within the TME, cancer-driven metabolic alterations such as nutrient competition and hypoxia reprogram endothelial PC composition, compromising vascular homeostasis. This leads to dysfunctional tumor vasculature characterized by reduced perfusion, enhanced leakiness, and an inflamed endothelial surface that supports immune cell adhesion and metastasis (Shi J. et al., 2025). Thus, endothelial PC remodeling emerges as a key regulatory axis that connects lipid metabolism to angiogenesis, immune modulation, and tumor progression.

## 4 Ferroptosis: a lipid-mediated cell death program

### 4.1 Ferroptosis mechanism

Ferroptosis is a distinct, iron-dependent form of regulated cell death that is mechanistically and morphologically divergent from apoptosis, necrosis, or autophagy. Its execution hinges on the convergence of three critical pathological conditions such as labile iron accumulation, reactive oxygen species (ROS) overload, and runaway lipid peroxidation of membrane PUFAs, particularly those esterified into phospholipids like phosphatidylcholine (Dixon et al., 2012; Panigrahi et al., 2022). Redox-active Fe^2+^ acts as a catalyst in Fenton reactions, generating hydroxyl radicals that initiate peroxidative chain reactions in PUFA-containing phospholipids. This leads to the accumulation of lipid hydroperoxides (LOOHs), which compromise membrane integrity, culminating in catastrophic cell lysis. The primary defense against oxidative damage is GPX4 a selenoenzyme that reduces LOOHs to non-toxic lipid alcohols using glutathione (GSH) as a cofactor. Ferroptosis is induced when this protective axis is disrupted either through direct pharmacological inhibition of GPX4 or indirectly via GSH depletion. The latter is frequently achieved by blocking cystine import through System xc^−^, thereby impairing GSH synthesis and compromising GPX4 activity (Bersuker et al., 2019) Importantly, ferroptosis is not a byproduct of oxidative stress but a tightly regulated death program, occur at the intersection of lipid metabolism, iron homeostasis, and redox regulation. It represents a potent therapeutic vulnerability in cancers characterized by metabolic rewiring and therapy resistance, especially those reliant on PUFA enriched membranes and compromised antioxidant capacity (Vani Raju et al., 2025).

### 4.2 Ferroptosis lipidomics

Ferroptosis is intimately administered by the composition of membrane phospholipids, especially the incorporation of PUFAs into key lipid classes such as phosphatidylethanolamine (PE) and PC. The susceptibility of a cell to ferroptosis is not dictated by total lipid content, but rather by its specific lipidomic signature, particularly the enrichment of phospholipids with PUFAs such as arachidonic acid (AA, C20:4) and adrenic acid (AdA, C22:4). These fatty acids contain bis-allylic hydrogen atoms that are chemically prone to radical abstraction, making them highly vulnerable to lipid peroxidation (Pope and Dixon, 2023). PE-PUFA species have been identified as primary executioners of ferroptotic death, although PC-PUFAs significantly contribute to the oxidative burden and serve as substrates for further propagation of lipid peroxidation. The biosynthesis of these oxidizable phospholipids is tightly regulated by enzymatic pathways involving ACSL4, which activates AA and AdA into acyl-CoA derivatives, and LPCAT3, which incorporates them into the sn-2 position of phospholipid backbones, especially PCs (Reed et al., 2022).

Once these PUFA-containing phospholipids are embedded in cellular membranes, they become substrates for oxidation via two primary mechanisms. One involves the enzymatic activity of lipoxygenases (LOX) such as ALOX15, which oxidize esterified PUFAs within phospholipids to generate lipid hydroperoxides (Ma X. H. et al., 2022). The other pathway is non-enzymatic autoxidation, wherein labile Fe^2+^ catalyzes the generation of ROS through Fenton chemistry, initiating peroxidation of PUFA chains Regardless of the initiating route, these oxidation events trigger a self-propagating lipid radical chain reaction, which, if not mitigated by GPX4, leads to membrane disruption and ferroptotic cell death. Therefore, the lipid composition of a cell specifically, the ratio of pro-ferroptotic PUFA-PEs and PUFA-PCs to protective monounsaturated fatty acids constitutes a decisive metabolic checkpoint that drives ferroptotic sensitivity. This lipidomic balance not only defines cellular fate under oxidative stress but also offers promising biomarkers and therapeutic targets in ferroptosis-driven cancer vulnerabilities (Dixon et al., 2012).

Thus, the ACSL4–LPCAT3 pathway functions as a key regulatory, integrating fatty acid metabolism with redox vulnerability. Its activity determines the membrane concentration of ferroptotic fuel and consequently, the intrinsic susceptibility of a cell to ferroptosis (Cui et al., 2023). Loss of function mutations or downregulation of either enzyme reduces PUFA incorporation into phospholipids and confers resistance to ferroptosis, further highlighting their essential roles in ordering ferroptotic commitment. This regulatory axis not only represents a critical control point in cell death signaling but also offers therapeutic leverage for selectively inducing ferroptosis in cancer cells with lipid metabolic dependencies.

### 4.3 Tumor sensitivity to ferroptosis

The therapeutic promise of ferroptosis lies in its selective cytotoxicity toward specific cancer subtypes, particularly those characterized by aggressive behavior, dedifferentiation, and resistance to conventional therapies. Tumor cell susceptibility to ferroptosis is not uniform but varies significantly across cancer types, reflecting differences in lipid metabolism, antioxidant capacity, and cellular phenotype. Mesenchymal-like tumor cells, which often arise via epithelial to mesenchymal transition (EMT), are prone to ferroptotic sensitivity. EMT is a developmental program co-opted in cancer to promote invasion, metastasis, and drug resistance. Mechanistically, EMT associated transcription factors such as ZEB1 not only repress epithelial gene expression but also enhance lipid remodeling by upregulating enzymes like ACSL4 and LPCAT3, while downregulating components of the antioxidant machinery, including GPX4 and glutathione biosynthesis pathways (Lin et al., 2023; Chauhan et al., 2025). This dual modulation increases the pool of oxidizable PUFA-phospholipids and weakens cellular defenses, priming mesenchymal cells for ferroptotic death.

Moreover, ferroptosis sensitivity is elevated in therapy-resistant cancer subpopulations, such as drug-tolerant persister (DTP) cells, which survive initial treatment by entering a quiescent, adaptive state. While these cells typically evade apoptosis through upregulation of survival proteins like BCL-2, their adaptation to therapeutic stress often induces oxidative imbalance and metabolic rewiring, including increased dependency on PUFA metabolism and redox regulation. These cells, while impervious to apoptosis-inducing therapies, remain vulnerable to lipid peroxidation, making ferroptosis a critical backdoor mechanism for eliminating residual, relapse-initiating cells (Lei et al., 2022). Cancers such as triple-negative breast cancer, renal cell carcinoma, and hepatocellular carcinoma have been shown to exhibit ferroptotic vulnerability, especially under oxidative stress or when antioxidant pathways are pharmacologically inhibited. The dependency on GPX4 the central lipid hydroperoxide detoxifying enzyme is not uniformly distributed across cancers, but is markedly amplified in tumors bearing specific oncogenic alterations that destabilize redox homeostasis. Among the most well-characterized are KRAS-mutant and p53-deficient tumors, where ferroptosis resistance becomes tightly coupled to metabolic reprogramming and antioxidant addiction.

**FIGURE 3:**
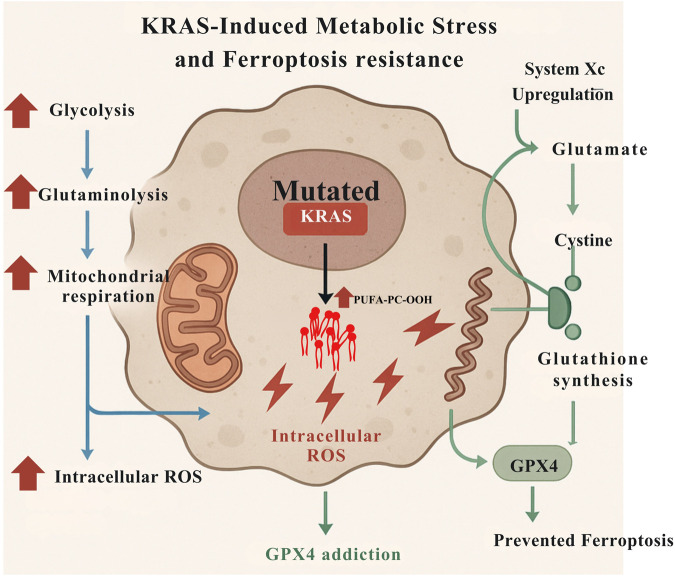
KRAS-driven oncogenic signaling induces metabolic reprogramming and ferroptosis resistance in pancreatic ductal adenocarcinoma cells. KRAS-mutant PDAC cells exhibit a hypermetabolic state characterized by enhanced glycolysis, glutaminolysis, and mitochondrial respiration, leading to elevated intracellular reactive oxygen species (ROS). This oxidative stress promotes lipid peroxidation, a key trigger of ferroptosis. To counteract this pro-oxidant environment, KRAS-mutant cells upregulate antioxidant defense mechanisms, including the cystine/glutamate antiporter System x_c^−^ (SLC7A11), glutathione biosynthesis, and glutathione biosynthesis, and glutathione peroxidase 4 (GPX4). GPX4 detoxifies lipid peroxides, preventing ferroptotic collapse and establishing a dependency on its continuous activity. The figure illustrates the metabolic and redox adaptations that enable KRAS-mutant cells to evade ferroptosis, highlighting the therapeutic vulnerability of GPX4 addiction.

KRAS-driven oncogenesis imposes a hypermetabolic state, fueled by elevated glycolysis, glutaminolysis, and mitochondrial respiration, all of which contribute to excessive intracellular reactive oxygen species (ROS) production. To counterbalance this pro-oxidant state, KRAS-mutant cells upregulate antioxidant systems, particularly the System x_c^−^ glutathione–GPX4 axis, to prevent ferroptotic collapse. This adaptation renders them addicted to GPX4, as its continuous activity is required to suppress lipid peroxidation triggered by oncogene-induced oxidative stress (Figure 3) (Lim and Leprivier, 2019). In KRAS-mutant colorectal cancers, especially in male patients, elevated GPX4 expression correlates with poor prognosis and ferroptosis suppression, underscoring the clinical relevance of this metabolic dependency (Lim and Leprivier, 2019).

Parallel to this, loss of p53 function further amplifies GPX4 reliance. Wild type p53 can promote ferroptosis by transcriptionally repressing SLC7A11, the key subunit of the System x_c^−^ cystine/glutamate antiporter. By limiting cystine import, p53 reduces glutathione biosynthesis, thereby weakening GPX4’s ability to neutralize lipid hydroperoxides (Liu et al., 2020). In contrast, p53-deficient tumors exhibit constitutive SLC7A11 upregulation, leading to robust cystine uptake and glutathione accumulation. Although this augments antioxidant defense, it also creates a synthetic lethality but these cells become critically reliant on the System x_c^−^–GSH GPX4 axis due to their loss of broader p53-regulated stress responses (Li et al., 2023; Nguyen et al., 2024). This non-oncogene addiction establishes GPX4 not only as a redox buffer but as a flaw in tumors with compromised tumor suppressor function.

Preclinical models validate the therapeutic tractability of GPX4 inhibition in such genetic contexts. In KRAS/p53 double-mutant pancreatic tumors, targeted degradation of GPX4 selectively induces ferroptosis in cancer cells without affecting immune cell viability and notably enhances the efficacy of immune checkpoint blockade (Li et al., 2023). These findings position GPX4 as a context-specific survival factor, offering a precision oncology approach for tumors that are often refractory to standard treatments.The oncogenic pressures exerted by KRAS mutations and p53 loss converge to create a redox-fragile yet ferroptosis-resistant phenotype, highly dependent on GPX4. Therapeutic exploitation of this dependency through GPX4 inhibition or glutathione depletion represents a rational and selective strategy to eliminate resilient, genetically defined tumor subpopulations that evade both apoptosis and immune clearance.

### 4.4 Ferroptosis in stromal and immune cells

Ferroptosis is not limited to tumor cells; it significantly impacts non-malignant elements of the tumor microenvironment (TME) including stromal and immune cells. Among these, tumor-infiltrating lymphocytes (TILs) particularly cytotoxic CD8^+^ T cells are especially vulnerable to ferroptotic death, a phenomenon that undermines anti-tumor immunity and facilitates immune evasion. This susceptibility arises from the harsh metabolic constraints imposed by the TME, where nutrient availability is tightly regulated by tumor cells to their own advantage.

**FIGURE 4:**
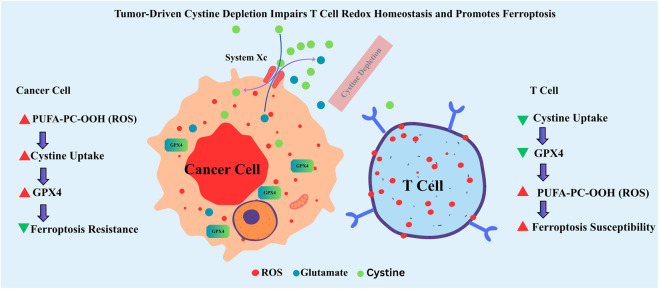
Tumor-induced cystine depletion promotes ferroptosis in T cells through glutathione deficiency and GPX4 inactivation. Cancer cells in the tumor microenvironment upregulate System x c^-^ to import cystine, a precursor for glutathione (GSH) synthesis. This metabolic competition reduces extracellular cystine levels, impairing GSH synthesis in nearby T cells. GSH is a critical cofactor for GPX4, which prevents ferroptosis by reducing lipid peroxides. In the absence of sufficient cystine, T cells fail to maintain GSH levels, resulting in GPX4 dysfunction and ferroptotic cell death. The figure illustrates the spatial and molecular dynamics of cystine competition, antioxidant depletion, and ferroptosis susceptibility in T cells within the tumor microenvironment.

A key mechanism driving T cell ferroptosis involves cystine deprivation in cancer cells that upregulate the System x_c^−^ (SLC7A11) antiporter to import cystine for glutathione (GSH) synthesis and maintenance of their antioxidant defenses. This metabolic competition depletes extracellular cystine, limiting its availability to neighboring T cells, which also rely on cystine for *de novo* GSH synthesis. GSH is the indispensable cofactor for GPX4, the central enzyme that detoxifies lipid hydroperoxides and prevents ferroptosis (Li et al., 2023). In cystine-limited conditions, T cells experience GSH depletion (Figure 4), rendering them incapable of neutralizing accumulating lipid peroxides and predisposing them to ferroptotic cell death.

Ironically, T cell activation itself amplifies this vulnerability. Upon antigen encounter, activated T cells undergo rapid membrane biogenesis enriched in PUFAs to support proliferation and effector functions. This lipid remodeling increases the abundance of peroxidation-prone phospholipids, thereby heightening susceptibility to ferroptosis precisely at the moment of maximal engagement with tumor cells (Ma et al., 2021). The resultant ferroptotic death selectively depletes the most functionally active T cells, thereby dismantling the immune response at its peak and blunting the effectiveness of immunotherapies like checkpoint inhibitors, which depend on the persistence of robust TIL populations. This tumor-induced ferroptosis in T cells constitutes a form of metabolic sabotage, where the cancer cell not only escapes immune destruction but actively induces the demise of its immune adversaries through nutrient competition and redox manipulation. Moreover, tumor-derived soluble factors such as exosomes and immunosuppressive cytokines may further inhibit cystine transporters on T cells or alter their metabolic state, compounding ferroptotic risk. Consequently, therapeutic strategies that protect T cells from ferroptosis such as enhancing GSH synthesis, stabilizing GPX4, or modulating PUFA incorporation could restore immune surveillance. On the other hand, encouraging tumor cell death and protecting immune effectors are two benefits of selectively inducing ferroptosis in tumor cells while avoiding TILs. Therefore, targeting ferroptosis in a cell-type-specific way may work in concert with immunotherapy to combat immune evasion and enhance clinical results (Srivastava et al., 2010).

Ferroptosis in cancer cells is not merely a terminal, cell-autonomous event characterized by lipid peroxidation and membrane rupture. Rather, it serves as a potent paracrine signaling mechanism that can dramatically influence the immunological architecture of the TME. The core execution mechanism unrestrained lipid peroxidation generates a distinct set of bioactive signals, including lipid hydroperoxides and their electrophilic degradation products, notably 4-hydroxynonenal (4-HNE) and malondialdehyde (MDA). These byproducts, traditionally viewed as mere hallmarks of oxidative damage, act as DAMPs that can modulate the phenotype and function of surrounding immune cells, particularly macrophages and dendritic cells (DCs) (Ayala et al., 2014).

Importantly, the immunological consequences of ferroptosis are highly context-dependent, demonstrating a dual-edged nature that may either stimulate or suppress anti-tumor immunity. On the one hand, ferroptosis can be immunogenic. Specific oxidized phospholipid species released from ferroptotic cells act as eat-me signals, enhancing the phagocytic uptake of dying cells by antigen-presenting cells such as DCs. This uptake facilitates DC maturation and promotes cross-presentation of tumor-associated antigens to CD8^+^ T cells, effectively priming adaptive anti-tumor immune responses (Bell et al., 2024). In this framework, ferroptosis aligns with the concept of immunogenic cell death (ICD), suggesting potential synergy with immune checkpoint inhibitors and cancer vaccines.

Conversely, the same class of electrophilic lipid mediators, particularly at high local concentrations, can exert immunosuppressive effects. Lipid aldehydes such as 4-HNE can directly impair lymphocyte viability and function, or covalently modify proteins, thereby disrupting key immune signaling pathways. Moreover, oxidized lipid species can polarize tumor-associated macrophages (TAMs) toward an M2-like phenotype, a state characterized by pro-angiogenic, tissue-repairing, and anti-inflammatory functions. This shift fosters immune tolerance and supports tumor progression, highlighting a paradox wherein ferroptotic death of cancer cells inadvertently reinforces the immunosuppressive barriers of the TME (Johnson et al., 2020). Therefore, the immunological output of ferroptosis depends on a complex interplay between the identity and concentration of released lipid mediators, the temporal dynamics of cell death, and the baseline immune contexture of the TME. Decoding this context-dependent signaling landscape is crucial for harnessing ferroptosis as a therapeutic modality, either as a direct anti-tumor strategy or to modulate immune responses in combination with immunotherapy.

## 5 Molecular crossroads: how PC metabolism regulates ferroptosis

### 5.1 PC as ferroptosis substrate reservoir

PUFAs such as AA and AdA incorporated into PC molecules confer heightened susceptibility due to their bis-allylic hydrogen atoms, which are especially prone to iron-catalyzed radical abstraction (Yaghmour et al., 2025). The specific enzymatic axis controlling PUFA incorporation into PC primarily the coordinated action of acyl-CoA synthetase long-chain family member 4 (ACSL4) and LPCAT3 acts as a metabolic gatekeeper of ferroptotic potential. ACSL4 activates free PUFAs into their CoA derivatives, while LPCAT3 facilitates their esterification into lysophosphatidylcholine backbones, ultimately generating peroxidation-prone PC-PUFA species (Reed et al., 2022). The upregulation of this enzymatic machinery in certain oncogenic contexts functionally transforms the otherwise inert PC pool into a pro-lethal lipid reservoir, primed for oxidation upon GPX4 inhibition or any perturbation in redox homeostasis.

The susceptibility of a cell to ferroptosis is intricately shaped by the enzymatic machinery regulating PC metabolism, with CHKA and LPCAT3 operating at two distinct yet converging nodes to fine-tune this vulnerability. CHKA, the rate-limiting enzyme of the *de novo* Kennedy pathway, primarily modulates the quantitative expansion of the PC pool. In cancer, CHKA is frequently upregulated under oncogenic control, driving the massive phospholipid biosynthesis required to support rapid proliferation and membrane biogenesis. While this hyperactivation serves as a core survival mechanism for tumor growth, it inadvertently intensifies ferroptotic vulnerability by fostering a cellular state that is heavily reliant on membrane integrity and robust redox defenses, particularly GPX4, to mitigate the lipid peroxidation burden that accompanies high metabolic throughput (Li et al., 2025). Thus, hyperactive CHKA signaling creates a metabolically primed but fragile architecture, where ferroptosis may be unleashed upon collapse of antioxidant buffering systems.

In contrast, LPCAT3 plays a more qualitative role in dictating ferroptotic susceptibility. As a critical enzyme in the Lands cycle, LPCAT3 selectively esterifies PUFAs particularly AA and AdA into the sn-2 position of lysophosphatidylcholine, thereby producing PUFA-enriched PC species that are highly prone to peroxidation (Reed et al., 2022). Elevated LPCAT3 activity results in a membrane lipid landscape enriched with ferroptosis-priming substrates, intensely increasing the risk of peroxidative collapse in the absence of GPX4 or glutathione. Conversely, suppression of LPCAT3 expression or function significantly attenuates ferroptosis by curtailing the formation of these oxidizable lipid species.

Together, CHKA and LPCAT3 form a coordinated metabolic circuit wherein CHKA supplies the bulk PC scaffold, and LPCAT3 dictates the oxidation potential of that scaffold by decorating it with PUFA chains. The balance between synthetic volume and remodeling specificity defines a molecular threshold for ferroptotic initiation. As such, the CHKA–LPCAT3 axis integrates membrane biogenesis with lipid remodeling, thereby modulating ferroptosis sensitivity in accordance with metabolic demand (Liu et al., 2025).

### 5.2 Phospholipid remodeling enzymes

The execution of ferroptosis is composed by a tightly regulated enzymatic network that directs the dynamic remodeling of membrane phospholipids conclusively shaping a cell susceptibility to lipid peroxidation. This network consists of a specialized cohort of phospholipid-modifying enzymes that manage both the incorporation of pro-ferroptotic PUFAs into membranes and the repair or removal of peroxidized lipids. At the lead of this priming phase is the sequential action of ACSL4 and LPCAT3. ACSL4 functions as a gatekeeper enzyme by selectively activating highly peroxidizable PUFAs, most notably AA and AdA into their acyl-CoA thioester derivatives, thereby determining the availability of substrates for membrane incorporation (Reed et al., 2022). This substrate specificity is a critical determinant of ferroptotic sensitivity, as cells deficient in ACSL4 are often resistant to ferroptosis.

Once activated, these PUFA-CoAs are utilized by LPCAT3, which catalyzes their esterification into LPC and lysophosphatidylethanolamine (LPE) backbones, ultimately enriching the PC and phosphatidylethanolamine (PE) pools with peroxidation-prone lipid species (Ma X. H. et al., 2022). This axis ACSL4–LPCAT3 thus serves as a metabolic channel for embedding ferroptosis-executing lipids into cellular membranes, effectively buildup the lipid bilayer with the biochemical substrates required for oxidative demise. Enhanced expression or activity of these enzymes has been consistently linked to increased ferroptosis sensitivity in both cancerous and non-transformed cells. In direct opposition to this pro-death remodeling machinery is the calcium-independent phospholipase A2 beta (PLA2G6, or iPLA2β), a membrane repair enzyme that mediates resistance to ferroptosis. PLA2G6 hydrolyzes the sn-2 acyl chain of peroxidized phospholipids, specifically recognizing and excising oxidatively damaged fatty acids from the membrane. This excision not only removes the oxidized moiety but also halts the propagation of the lipid peroxidation chain reaction, thereby constituting a vital intracellular defense mechanism against ferroptotic death (Beharier et al., 2020). Functionally, PLA2G6 may either attenuate ferroptosis by enabling lipid repair or under conditions of sustained oxidative stress become overwhelmed, with the released oxidized lipids themselves contributing to redox signaling or further injury. The kinetic competition between these opposing enzymatic forces PUFA incorporation via ACSL4–LPCAT3 and lipid peroxide excision via PLA2G6 ultimately determines whether a cell resists or capitulates to ferroptosis. When the rate of oxidizable phospholipid incorporation surpasses the enzymatic capacity for lipid repair and antioxidant defense, lipid peroxidation proceeds uncontrollably, culminating in membrane disruption and ferroptotic death (Liu et al., 2022a). This enzymatic tug-of-war not only defines the cell ferroptotic threshold but also presents a therapeutic window to manipulate ferroptosis for cancer therapy by targeting either arm of this lipid remodeling axis.

## 6 PC-ferroptosis axis as a mediator of tumor-stroma interactions

### 6.1 CAFs and lipid crosstalk

**FIGURE 5:**
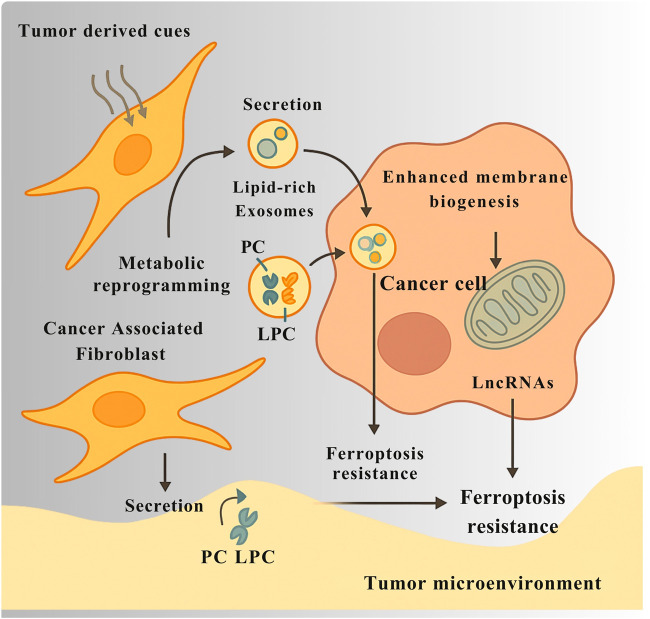
CAFs support ferroptosis resistance in cancer cells through metabolic and exosomal communication. In response to signals from tumor cells, cancer-associated fibroblasts (CAFs) change their metabolism and begin producing more lipids. They release exosomes packed with phosphocholine (PC), lysophosphatidylcholine (LPC), and long non-coding RNAs (LncRNAs). These exosomes deliver lipids that help cancer cells build membranes and produce energy, reducing their need to make these molecules themselves. The LncRNAs carried by the exosomes also change gene activity in cancer cells, helping them resist ferroptosis by lowering oxidative stress and lipid damage. Together, this exchange between CAFs and cancer cells creates a local environment that protects tumors from ferroptotic cell death.

The regulatory axis connecting PC metabolism to ferroptotic cell death extends beyond a cell-autonomous phenomenon, functioning instead as a critical interface in tumor-stroma crosstalk, with CAFs emerging as key non-malignant architects of ferroptotic resistance. Under the influence of tumor-derived cues, CAFs undergo extensive metabolic reprogramming, adopting an affluent phenotype that actively modulates the nutrient and redox landscape of the TME. A pivotal aspect of this reprogramming involves the secretion of lipids and release of exosomes enriched with PC and its derivative LPC. These CAF-derived exosomes act as a targeted nutrient shuttle, delivering preformed lipids that support membrane biogenesis and bioenergetics in adjacent cancer cells thus relieving them of *de novo* biosynthetic burden (Figure 5). This lipid transfer is functionally coupled to gene-regulatory cargo, as demonstrated by the delivery of long non-coding RNAs via CAF-derived exosomes, which enhance ferroptosis resistance in recipient tumor cells (Ye et al., 2023).

Beyond passive metabolic support, CAFs play an active cytoprotective role, erecting a non-cell-autonomous antioxidant shield that protects tumor cells from lipid peroxidation and ferroptotic death. One of the most direct mechanisms of this stromal intervention is the secretion of glutathione (GSH), a critical cofactor for GPX4 into the extracellular space. In colorectal cancer models, this stromal GSH is imported by cancer cells, reinforcing intracellular antioxidant defenses and conferring resistance to ferroptosis inducers such as erastin and RSL3. In parallel, specific CAF subpopulations establish an inflammatory signaling environment that triggers NRF2 pathway activation in neighboring tumor cells, leading to transcriptional upregulation of antioxidant genes and further shielding the lipidome from peroxidation-induced demise (Ma F. et al., 2022). Collectively, these findings position CAFs not merely as passive bystanders but as active sentinels of redox balance, functioning through lipid transfer, antioxidant donation, and transcriptional reprogramming to maintain a ferroptosis-resistant niche. This stromal transposition of ferroptotic resistance presents a formidable obstacle to the clinical exploitation of ferroptosis-inducing therapies and underscores the necessity of co-targeting CAF-derived protective mechanisms to achieve effective tumor eradication.

### 6.2 Immune cells and ferroptosis signals

**FIGURE 6:**
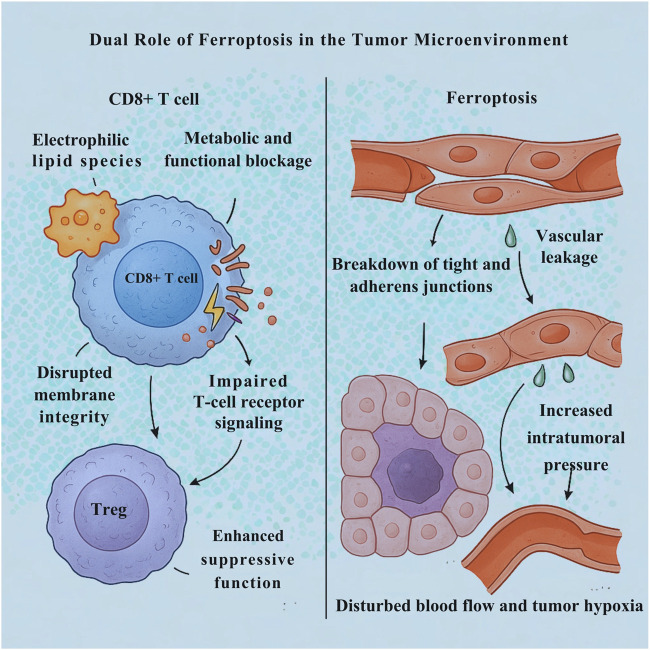
Ferroptosis applies a dual pathological influence within the tumor microenvironment (TME), simultaneously impairing immune function and destabilizing vascular architecture. Electrophilic lipid species generated during ferroptosis covalently modify proteins essential for CD8^+^ T-cell receptor signaling, leading to membrane disruption, bioenergetic paralysis, and T-cell exhaustion, while also enhancing the suppressive stability of regulatory T cells (Tregs), thereby reinforcing immune evasion. In parallel, ferroptosis in endothelial cells (ECs) disrupts tight and adherens junctions, resulting in vessel wall breakdown, vascular leakage, and increased intratumoral pressure. This hemodynamic disarray impairs tumor hypoxia by impairing oxygen delivery.

The immunological effects of ferroptosis are coordinated by the paracrine bioactivity of its released molecular components, which reshape the immunological architecture of the TME and extend far beyond the death of individual cancer cells. As ferroptosis progresses, PUFA–PC species as previously described in Section 5.1 as pre-existing substrates within cellular membranes undergo stepwise oxidation driven by iron-dependent lipid peroxidation. These oxidized derivatives gradually accumulate as oxidized phosphatidylcholine (oxPC) intermediates, which alter membrane structure and redox homeostasis even before full cell lysis. Upon ferroptotic membrane rupture, these oxPC species, along with reactive aldehydes such as 4-hydroxynonenal (4-HNE) and malondialdehyde (MDA), are released into the extracellular space, where they function as potent damage-associated molecular patterns (DAMPs). These lipid-derived signals activate pattern recognition receptors including TLR2, TLR4, and CD36, stimulating cytokine production, macrophage polarization, and dendritic cell activation. Thus, PUFA–PC and oxPC represent sequential biochemical states within the ferroptotic cascade transitioning from structural membrane substrates to immunogenic mediators that link intracellular lipid peroxidation to extracellular immune modulation within the TME. These oxidized lipids are sensed by innate immune sentinels, including macrophages and DCs, initiating downstream signaling cascades that drive context-specific immunological responses. Under certain conditions, these DAMPs can trigger a robust immunogenic response, particularly when they are taken up by DCs, leading to enhanced antigen processing and cross-presentation, DC maturation, and the activation of tumor-specific CD8^+^ T cells. Such events categorize ferroptosis as a form of ICD capable of amplifying adaptive anti-tumor immunity (Wiernicki et al., 2022). However, this pro-immunogenic axis is far from universal. In the setting of an established or immunosuppressive TME, ferroptotic DAMPs often promote the opposite outcome. The same lipid peroxidation products can drive macrophage polarization toward the M2-like, wound-healing phenotype, characterized by the secretion of anti-inflammatory cytokines, promotion of extracellular matrix remodeling, and suppression of cytotoxic effector functions (Vassiliou and Farias-Pereira, 2023) as schematically represented in Figure 4. Moreover, the accumulation of oxidized lipids within the TME imposes a direct metabolic and functional blockade on CD8^+^ T cells, impairing membrane integrity, disrupting key signaling pathways, and driving T-cell exhaustion (Figure 6). These electrophilic lipid species can covalently modify proteins essential for T-cell receptor signaling, leading to a state of bioenergetic paralysis and diminished effector capacity. Compounding this immune dysfunction, emerging evidence suggests that ferroptotic lipid mediators can enhance the stability and suppressive function of regulatory T cells (Tregs), further reinforcing the immunosuppressive firewall that protects the tumor from immune attack (Vassiliou and Farias-Pereira, 2023). Collectively, these findings highlight the immunological duality of ferroptosis capable of either igniting tumor immunity and strengthening immune escape, depending on the prevailing microenvironmental cues (Jiang et al., 2019). Understanding and therapeutically modulating this ferroptosis immune axis is thus crucial. Targeted manipulation of lipid DAMP signaling, macrophage polarization, or T-cell susceptibility to oxidized lipids may unlock the potential of ferroptosis-inducing agents not just as cytotoxic therapies, but as immune modulators capable of tipping the TME toward durable anti-tumor immunity.

### 6.3 Endothelial cell ferroptosis and vascular integrity

The tumor-stroma dialogue extends to the very vasculature that sustains the malignant mass, with the ferroptotic vulnerability of endothelial cells emerging as a pivotal regulator of vascular function and, by extension, the immune accessibility of TME. Ferroptosis in endothelial cells (ECs) represents a double-edged therapeutic strategy while it can disturb tumor perfusion by targeting the vessel lining, this same process risks compromising vascular integrity. Ferroptotic EC death leads to disintegration of tight and adherens junctions, culminating in vessel wall breakdown. The resulting vascular leakage allows plasma proteins and interstitial fluid to flood the tumor stroma, raising intratumoral pressure and disorganizing blood flow. This hemodynamic chaos precipitates a marked worsening of tumor hypoxia, as oxygen delivery becomes increasingly inadequate. Such hypoxia-driven vascular collapse is not merely a metabolic consequence also it actively enforces immune exclusion, creating a physicochemical barrier that prevents the infiltration and effector function of cytotoxic T lymphocytes (CTLs). The tumor core, thus deprived of immune surveillance, becomes an immunologically privileged niche, resistant to immune checkpoint inhibitors and other immunotherapies (Bakleh and Al Haj Zen, 2025).

The susceptibility of endothelial cells to ferroptotic collapse is intrinsically tied to their membrane lipid composition, particularly the dynamic remodeling of PC. The ACSL4–LPCAT3 axis, by esterifying PUFAs like arachidonic acid into PC, generates a pro-ferroptotic lipid milieu. Conversely, membranes enriched in monounsaturated fatty acids (MUFAs) confer resistance to lipid peroxidation. This balance governs membrane fluidity and mechanosensitivity, which are essential for responding to shear stress, maintaining eNOS localization, and preserving vascular tone (Esposito et al., 2024; Bakleh and Al Haj Zen, 2025). When this balance tips toward PUFA enrichment, ECs become primed for ferroptosis, linking endothelial metabolism to vascular fragility. Targeting endothelial ferroptosis, therefore, offers a paradoxical therapeutic opportunity. While uncontrolled induction may exacerbate hypoxia and immune escape, strategic modulation could help normalize the tumor vasculature restoring perfusion, relieving hypoxia, and facilitating immune cell infiltration. This nuanced approach positions endothelial ferroptosis as a vascular checkpoint that determines the functional accessibility of tumors to both oxygen and immunity and highlights the need for temporal and spatial precision in ferroptosis-targeted interventions (Wang-Bishop et al., 2023).

### 6.4 Exosomes and lipid exchange

The regulation of ferroptosis sensitivity extends beyond intrinsic cellular properties or direct intercellular contact, encompassing a complex and highly dynamic form of intercellular lipid communication mediated by EVs, particularly exosomes. These nano-sized vesicles, secreted by virtually all cells within the TME, serve as lipid-laden messengers that traffic critical components of the PC metabolic axis between diverse stromal and malignant cell populations. Notably, tumor-derived exosomes are enriched in LPC, oxidized phospholipids, and lipid peroxidation products, reflecting the metabolic and redox status of the donor cell and thereby functioning as a snapshot of ferroptotic readiness (Ma et al., 2024). This vesicle-mediated lipid transfer has profound functional implications andit allows for the non-cell-autonomous modulation of ferroptotic susceptibility. For instance, exosomes released by ferroptosis-resistant cancer cells can deliver lipid species or redox buffers that confer protection to more vulnerable neighbors. Additionally, exosomal cargo may include regulatory RNAs such as miRNAs or long non-coding RNAs that, once internalized by recipient cells, suppress the expression of key pro-ferroptotic genes such as ACSL4 or ALOX15, thereby lowering ferroptosis sensitivity across the local cellular network. Beyond cancer cells themselves, CAFs also participate in this vesicle-based metabolic buffering as illustrated in Figure 5, which depicts the transfer of lipid-rich exosomes and antioxidant molecules from stromal to tumor cells that reinforce ferroptosis resistance within the tumor microenvironment. CAF-derived exosomes can be loaded with lipids, cholesterol, or antioxidant molecules, which recipient tumor cells can incorporate into their own membranes or metabolic pathways to mitigate lipid peroxidation stress. In doing so, CAFs act as metabolic support hubs, promoting a ferroptosis-resistant ecosystem that facilitates tumor survival under oxidative and nutrient-deprived conditions (Xiao et al., 2024). Beyond exosome-mediated lipid exchange, recent evidence highlights tunneling nanotubes (TNTs) as an additional, direct mechanism of intercellular communication within the tumor microenvironment. These actin-based cytoplasmic bridges physically connect adjacent cells, enabling the transfer of mitochondria, lipid droplets, and signaling molecules that modulate bioenergetic adaptation and redox balance. Through TNTs, cancer cells can acquire functional mitochondria from stromal or immune cells, restoring oxidative metabolism under stress conditions and enhancing therapy resistance (Feng et al., 2025). Moreover, the exchange of arachidonic acid and other lipid mediators via TNTs contributes to metabolic coupling between tumor and stromal compartments, complementing the vesicle-based metabolic buffering provided by exosomes. Integrating TNT biology into the framework of lipid-mediated communication provides a more comprehensive understanding of how tumors coordinate metabolic and redox homeostasis to sustain growth and evade ferroptotic stress (Allegra et al., 2022). These findings position exosomes not merely as byproducts of cellular turnover, but as functional units of lipidomic and redox regulation that coordinate ferroptosis susceptibility at the community level. By distributing lipid species, modulating gene expression, and equalizing oxidative burden, exosomes help normalize ferroptotic risk across the tumor-stroma interface, highlighting vesicle trafficking as both a mechanism of tumor adaptation and a potential therapeutic target to disrupt ferroptosis resistance circuitry in cancer.

## 7 Therapeutic implications and interventions

### 7.1 Targeting PC metabolism

The centrality and pleiotropy of PC metabolism in sustaining the malignant phenotype establish its enzymatic pathways as highly tractable targets for therapeutic intervention. Cancer cells exhibit profound dependency on PC-related processes for membrane biogenesis, oncogenic signaling, and metabolic crosstalk with the stroma, rendering this axis a metabolic vulnerability. One of the most direct and extensively explored strategies involves inhibiting the *de novo* Kennedy pathway, particularly by targeting its rate-limiting enzyme, CHKA. Frequently overexpressed across diverse malignancies and strongly associated with poor clinical outcomes, CHKA serves as both a biomarker of aggressiveness and a therapeutic node. Small-molecule CHKA inhibitors, such as TCD-717, have shown promising anti-tumor activity in preclinical models by inducing a membranostatic crisis halting membrane phospholipid synthesis, impairing proliferative expansion, and precipitating metabolic collapse through disrupted lipid homeostasis (Liu et al., 2025). In parallel, post-synthetic remodeling of PC represents a complementary target space. Inhibiting enzymes such as LPCAT3, which incorporates PUFAs like arachidonic acid into PC, could theoretically limit the generation of peroxidation-prone phospholipids and dampen ferroptosis susceptibility. However, this approach presents a inconsistency while it may reduce oxidative stress burden, it could also render cancer cells more resistant to ferroptosis-inducing agents, demanding careful therapeutic context and combinatorial logic in clinical applications (Reed et al., 2022). An increasingly promising walk targets the extracellular conversion of LPC to lysophosphatidic acid (LPA) by autotaxin (ATX; ENPP2). This secreted lysophospholipase D is often overexpressed by both tumor and stromal cells and plays a pivotal role in fueling paracrine LPA signaling, which activates PI3K/AKT, MAPK, and Rho pathways to promote tumor proliferation, angiogenesis, immune evasion, and metastasis. Unlike intracellular lipid enzymes, ATX’s extracellular activity renders it highly druggable, and several ATX inhibitors are advancing through clinical development pipelines. These compounds offer a direct route to disrupt lipid-based tumor-stroma communication, shutting down a key mitogenic and pro-invasive feedback loop (Valdés-Rives and González-Arenas, 2017). Collectively, these multi-pronged therapeutic strategies reflect a shift from targeting tumor cells in isolation toward systemically dismantling the lipid infrastructure that reinforces cancer growth and resilience. By impairing membrane production, rewiring ferroptotic sensitivity, and silencing lipid-mediated paracrine signaling, PC metabolism-targeted therapies hold promise not just as direct anti-neoplastics, but as master regulators of the tumor ecosystem.

### 7.2 Ferroptosis-inducing drugs

The therapeutic induction of ferroptosis has emerged as a promising strategy to selectively eradicate metabolically stressed tumor cells, with Class 1 inducers representing a mechanistically distinct class that disrupts the antioxidant defense machinery upstream of GPX4 activity. These agents function by targeting the biosynthetic supply of glutathione (GSH), the essential reducing cofactor for GPX4, which is the central enzymatic barrier against lipid peroxidation. The principal molecular target of Class 1 inducers is System xc^−^, a sodium-independent plasma membrane antiporter composed of SLC7A11 (xCT) and SLC3A2, which mediates the import of extracellular cystine in exchange for intracellular glutamate (Dixon et al., 2012). Erastin, the prototypical Class 1 ferroptosis inducer, and its optimized derivatives act by inhibiting System xc^−^, thereby blocking the influx of cystine the rate-limiting substrate for intracellular GSH biosynthesis. Upon cellular entry, cystine is reduced to cysteine, a process that is essential for maintaining adequate GSH levels. Inhibiting this transport step leads to acute GSH depletion, thereby collapsing the redox buffering capacity of the cell and functionally inactivating GPX4, which relies on GSH to catalytically detoxify membrane-associated lipid hydroperoxides (Perera et al., 2025). Importantly, this approach does not target GPX4 directly but instead starves it of its functional substrate, rendering the enzyme catalytically inert and exposing the membrane to unmitigated lipid peroxidation. This indirect yet potent strategy is particularly effective against tumors with heightened dependency on antioxidant systems, a metabolic phenotype commonly seen in cancers with oncogenic activation MYC, KRAS or loss of tumor suppressors such as p53. Figure 3 schematically illustrates this dependency, highlighting how these oncogenic alterations enhance reliance on the GPX4-centered redox network to maintain ferroptosis resistance. Wild-type p53 is known to transcriptionally repress SLC7A11, and loss-of-function p53 mutations result in its upregulation, creating a compensatory reliance on System xc^−^ that can be exploited therapeutically (Liu et al., 2020). Thus, Class 1 ferroptosis inducers exploit a form of synthetic lethality, targeting the metabolic addiction of cancer cells to cystine uptake and antioxidant maintenance. By collapsing this redox defense axis, they effectively tip the balance toward iron-dependent lipid peroxide accumulation and ferroptotic death, representing a precision-guided approach to subvert tumor redox homeostasis.

In contrast to the upstream metabolic blockade imposed by Class 1 ferroptosis inducers, Class 2 agents act at the core of the ferroptotic machinery, executing cell death through direct and irreversible inhibition of GPX4. GPX4 is a selenoenzyme uniquely capable of reducing membrane embedded lipid hydroperoxides to their corresponding alcohols, thereby preventing the propagation of oxidative damage that would otherwise lead to membrane rupture. Class 2 inducers including the archetypal compounds RSL3 and ML210 were identified through synthetic lethality screens for molecules selectively lethal to oncogenic RAS- expressing cells, establishing their potential in targeting redox-addicted malignancies (Perera et al., 2025). Mechanistically, these compounds function as electrophilic warheads that covalently modify the active site selenocysteine of GPX4. This adduction permanently inactivates GPX4, crippling the cell last line of defense against iron-catalyzed lipid peroxidation. As a result, even basal levels of lipid ROS, normally buffered by GPX4, become sufficient to initiate an unrestrained peroxidation chain reaction, culminating in catastrophic membrane destabilization and ferroptotic cell death (Gu et al., 2025). A defining pharmacological attribute of Class 2 inducers is their glutathione-independent mechanism of action. Unlike Class 1 agents like erastin, whose efficacy depends on glutathione depletion via System xc^−^ inhibition, Class 2 compounds bypass upstream cystine metabolism entirely, directly targeting the execution point of ferroptosis. This renders them predominantly effective in contexts where cancer cells have acquired resistance to cystine deprivation, such as through alternative amino acid transporters, upregulated transsulfuration pathways, or compensatory antioxidant networks (Du and Guo, 2022). Thus, Class 2 inducers provide a vigorous and extraneous strategy to initiate ferroptosis, especially in tumors that have evolved resistance to metabolic stress-based therapies. By directly disabling the enzymatic constraint on ferroptotic execution, they ensure irreversible lipid peroxide accumulation, positioning GPX4 inhibition as a powerful and non-redundant entry point for therapeutic intervention in redox-driven malignancies. Beyond the canonical inhibitors of System xc^−^ and GPX4, a third category of compounds induces ferroptosis by promoting a pro-oxidant lipid environment through more diverse and sometimes unique mechanisms. This class includes repurposed clinical drugs and novel experimental agents that dismantle cellular antioxidant defenses or directly facilitate lipid peroxidation. Sulfasalazine, an FDA-approved drug with a long history of use as an anti-inflammatory agent, has been identified as a potent ferroptosis inducer. While its chemical structure is distinct from erastin, its primary mechanism of action in this context is convergent with Class 1 inducers as it functions as a competitive inhibitor of the System xc^−^ antiporter. By blocking cystine uptake, sulfasalazine similarly manages the depletion of intracellular glutathione, thereby sensitizing cancer cells to ferroptosis (Jia et al., 2025). Its clinical availability and established safety profile have made it an important tool for investigating the therapeutic potential of ferroptosis induction in a more translational setting. A more mechanistically distinct compound is FIN56, which induces ferroptosis through a sophisticated, dual-pronged attack that evades direct inhibition of GPX4 or System xc^−^. First, FIN56 promotes the degradation of the GPX4 protein itself through a process dependent on acetyl-CoA carboxylase (ACC) and the subsequent synthesis of specific lipid species. This represents a fundamentally different strategy from the direct covalent inactivation achieved by Class 2 inhibitors like RSL3 instead of simply disabling the enzyme, FIN56 eliminates it from the cell entirely. Second, and in parallel, FIN56 leads to the depletion of coenzyme Q10 (CoQ10), a crucial lipophilic radical-trapping antioxidant that functions as a key component of the FSP1-CoQ10-NAD(P)H axis, an independent anti-ferroptotic system that operates in parallel to the GPX4 pathway. By simultaneously dismantling the two primary defenses against lipid peroxidation removing the GPX4 protein and depleting the CoQ10 shield FIN56 powerfully shifts the cellular redox balance toward a catastrophic, pro-ferroptotic state (Bersuker et al., 2019). Together, agents like sulfasalazine and FIN56 illustrate the rich diversity of druggable nodes within the ferroptosis network, demonstrating that lipid peroxidation can be triggered not only by disabling the core antioxidant machinery but also by targeting its substrate supply or by inhibiting parallel protective pathways.

### 7.3 Synergistic strategies

The intricate metabolic wiring of cancer cells presents a rational framework for developing synergistic therapeutic strategies, wherein the simultaneous targeting of distinct but interconnected pathways can achieve a therapeutic effect far greater than the sum of the individual agents. A particularly promising approach involves combining inhibitors of PC metabolism with direct ferroptosis inducers. This combination paradigm is based on the principle of a one-two punch the first agent acts to prime the cancer cell by inducing metabolic stress and creating a sharp state of vulnerability, while the second agent delivers the lethal ferroptotic blow by disabling the already-strained antioxidant defenses. For example, the use of a CHKA inhibitor disrupts the *de novo* synthesis of PC, leading to membrane stress, ER stress, and a general state of metabolic disarray. This does not necessarily kill the cell outright but places it in a precarious state of sensitive oxidative stress, increasing its reliance on the GPX4 pathway for survival. By subsequently administering a ferroptosis inducer such as erastin to deplete glutathione or RSL3 to directly inhibit GPX4 it becomes possible to overcome this overburdened defense system using doses that might be sub-lethal when used as a monotherapy (Park et al., 2023). This synthetic lethal approach holds the potential to overcome both intrinsic and acquired resistance to therapy. Cancer cells that can compensate for the metabolic attack of a CHKA inhibitor may not be able to withstand the concurrent loss of their primary antioxidant shield. Conversely, cells with healthy GPX4 activity that makes them resistant to ferroptosis inducers may become susceptible once their overall metabolic integrity is compromised by the inhibition of PC synthesis. The principle extends beyond CHKA by combining inhibitors of autotaxin with pro-ferroptotic drugs could also be highly synergistic. By blocking the pro-survival, mitogenic signaling generated by the ATX-LPA axis, the intrinsic apoptotic threshold is lowered, potentially making it more vulnerable to the membrane damage inflicted by ferroptosis (Laface et al., 2024). Furthermore, this logic can be broadened to include standard-of-care treatments like radiation and certain chemotherapies, which themselves induce massive oxidative stress as a primary mechanism of action. Co-administering these therapies with a ferroptosis inducer systematically dismantles the cell ability to cope with that oxidative stress is a highly rational strategy for achieving profound radiosensitization and chemosensitization, offering a powerful avenue to enhance the efficacy of established cancer treatments (Zeng et al., 2023). A supreme challenge in harnessing the therapeutic potential of ferroptosis lies in navigating its complex and often paradoxical effects on the immune system. While ferroptosis can be immunogenic under certain conditions, the concurrent risk of fostering an immunosuppressive TME necessitates a rationally designed combination strategy. A highly promising approach involves the combination of ferroptosis-inducing drugs with immune checkpoint inhibitors (ICIs), such as antibodies targeting the PD-1/PD-L1 axis. The primary rationale for this synergy is twofold and first, to leverage the immunogenic potential of ferroptosis to ignite an anti-tumor immune response, and second, to use ICIs to simultaneously overcome the potential for ferroptosis-mediated immunosuppression. As ferroptotic cancer cells release their DAMPs and tumor-associated antigens, they create an opportunity to prime an adaptive immune response. However, this process can also trigger compensatory upregulation of immunosuppressive checkpoints like PD-L1 on both surviving cancer cells and tumor-infiltrating myeloid cells, effectively creating a braking mechanism that reduces the nascent immune attack. The concurrent administration of an anti-PD-L1 antibody can dismantle this adaptive resistance, releasing the brakes on T-cells and allowing them to fully engage with and eliminate the antigen-presenting tumor cells (Tewari et al., 2021). Furthermore, this combination directly addresses the issue of T-cell vulnerability to ferroptosis within the metabolically hostile TME. By inducing ferroptosis in a large number of cancer cells, it may be possible to transiently alleviate the intense metabolic competition for key nutrients like cystine, thereby creating a more permissive environment for T-cell function. More importantly, the administration of ICIs reinvigorates exhausted T-cells, potentially enhancing their metabolic fitness and their own antioxidant capacity, making them more resilient to the immunosuppressive lipid DAMPs released during tumor cell death (Li H. et al., 2022). Several preclinical studies have provided compelling proof-of-concept for this approach, demonstrating that combining a ferroptosis inducer with PD-1/PD-L1 blockade leads to superior tumor control compared to monotherapy alone. This synergistic effect is often dependent on CD8^+^ T-cells, confirming that the therapeutic benefit is immunologically driven. This strategy, therefore, represents a sophisticated interplay where ferroptosis acts to inflame the tumor and reveal its antigens, while ICIs ensure that the responding immune system has the sustained capacity to execute its anti-tumor program effectively, transforming a potentially immunosuppressive cell death event into a powerful adjuvant for cancer immunotherapy (Ju et al., 2022).

### 7.4 Biomarkers and diagnostics

The clinical translation of therapies that either induce or are modulated by ferroptosis is critically dependent on the development of robust biomarkers to identify susceptible patient populations, monitor therapeutic response, and guide combination strategies. A multi-tiered approach encompassing lipidomics, genomics, and advanced imaging is emerging to meet this need. At the most fundamental level, the cellular lipidomic signature provides a direct readout of a tumor’s intrinsic susceptibility. Mass spectrometry-based analysis of tumor tissue or even circulating lipids can reveal specific metrics, such as the ratio of PC to phosphatidylethanolamine (PE) or the ratio of LPC to PC, which can reflect the rate of PC turnover and remodeling. More specifically, quantifying the abundance of phospholipids containing highly peroxidizable polyunsaturated fatty acids like arachidonic and adrenic acid offers a direct measurement of the available fuel for ferroptosis, providing a powerful predictive biomarker for sensitivity to agents like RSL3 (Peng et al., 2021). Complementing this lipid-centric view is the analysis of ferroptosis-related gene expression. Transcriptomic or proteomic profiling of tumor biopsies can quantify the expression levels of key genes that govern ferroptosis sensitivity, effectively mapping the state of the regulatory network. High expression of pro-ferroptotic genes like ACSL4, combined with low expression of the primary defense enzyme GPX4, would strongly predict a high intrinsic vulnerability. Conversely, the high expression of the cystine importer SLC7A11 could indicate a dependency on this pathway, predicting sensitivity to Class 1 ferroptosis inducers like erastin while also suggesting potential resistance to oxidative therapies that the cell is well-equipped to handle (Battaglia et al., 2025). A composite ferroptosis score based on the weighted expression of a panel of such genes could offer a clinically actionable metric to guide patient selection. Finally, the non-invasive imaging of ferroptosis *in vivo* represents a crucial Frontier for monitoring real-time therapeutic response. Advanced imaging techniques are being developed to visualize the key biochemical events of ferroptosis. Redox-sensitive Magnetic Resonance Imaging (MRI) probes can detect shifts in the tissue redox state, providing an indirect measure of the oxidative stress induced by therapy. More directly, the development of novel positron emission tomography (PET) radiotracers targeting key proteins like SLC7A11 is underway to spatially map ferroptosis sensitivity within a patient’s tumors (Kato et al., 2025). At the cellular level, significant progress has been made in creating highly specific fluorescent probes like C11-BODIPY can directly detect lipid peroxidation and visualize oxidized PCs in preclinical models. While translating these high-resolution imaging agents to the clinic remains a challenge, these innovations collectively signal a move towards a new era of precision medicine where the ferroptotic state of a tumor can be dynamically assessed, enabling the rational deployment of therapies designed to exploit this unique cell death pathway (Drummen et al., 2002; Dai et al., 2023).

## 8 Challenges and future directions

### 8.1 Heterogeneity in PC/Ferroptosis regulation

A primary challenge impeding the broad clinical application of therapies targeting PC metabolism and ferroptosis is the profound heterogeneity that characterizes TME. This variability exists on multiple interconnected levels, demanding a far more significant approach than one size that fits all strategy. The most significant is the considerable interpatient and intertumoral variability in lipid metabolism. Tumors arising from different tissues, and even histologically similar tumors from different patients, exhibit vastly different metabolic landscapes, dictated by their unique combination of genetic drivers, epigenetic state, and the selective pressures of their microenvironment. A cancer driven by MYC may be intricately dependent on the CHKA-mediated *de novo* synthesis pathway for PC, whereas another might primarily rely on lipid scavenging or possess an intrinsically healthy antioxidant system. This inherent variability translates directly into a heterogeneous ferroptotic potential, where a tumor’s baseline expression of key regulators like ACSL4 and GPX4 can render it either sensitive or profoundly resistant to pro-ferroptotic agents before any therapy is administered (Jia et al., 2020). This complexity is magnified exponentially by the intratumoral heterogeneity of the stromal compartments, which have unique and highly dynamic metabolic roles. TME is not a homogenous mixture, but a spatially organized ecosystem composed of functionally distinct subpopulations of cells, each with its own metabolic program. For instance, the cancer-associated fibroblast (CAF) population is not monolithic different CAF subtypes can coexist within a single tumor, some of which may actively secrete protective lipids and antioxidants that shield cancer cells from ferroptosis, while others may not. This spatial arrangement of metabolic symbiosis and antagonism means that a therapy efficacy may depend entirely on a cancer cell proximity to a protective versus a neutral stromal cell (Kalluri, 2016). Furthermore, these stromal roles are dynamic and plastic, capable of changing in response to therapy or evolving tumor signals. The same dynamism applies to immune cells, whose own metabolic reprogramming and vulnerability to ferroptosis are dictated by the local nutrient and redox environment shaped by the entire cellular community. Therefore, a major future direction must involve moving beyond a cancer cell centric view towards single-cell, spatially resolved analyses that can map this complex metabolic interplay. Overcoming this challenge will require the development of sophisticated, biomarker-driven strategies capable of accounting for the metabolic state of the entire tumor ecosystem to rationally predict and overcome therapeutic resistance (Sun et al., 2019).

### 8.2 Technical hurdles

A significant barrier to fully translating our growing understanding of PC metabolism and ferroptosis into clinical practice is the presence of formidable technical hurdles associated with interrogating these processes with the necessary precision and spatiotemporal resolution. Conventional bulk analysis techniques, such as liquid chromatography-mass spectrometry (LC-MS) applied to homogenized tumor tissue, have been instrumental in defining the fundamental lipidomic signatures associated with ferroptosis sensitivity. However, these methods average the signals from millions of cells cancer, stromal, and immune and thus completely obscure the critical spatial relationships and intercellular heterogeneity that define the TME. To move forward, there is a profound need for advanced spatial lipidomics, techniques that merge the chemical specificity of high-resolution mass spectrometry with spatial information. Methods like matrix-assisted laser desorption/ionization mass spectrometry imaging (MALDI-MSI) and secondary ion mass spectrometry (SIMS) are at the forefront of this effort, enabling the mapping of specific PC species, their oxidation products, and even pathway inhibitors directly onto tissue sections. This allows researchers to ask, for example, whether cancer cells adjacent to CAFs have a different lipid profile than those near blood vessels, providing an unprecedented view of metabolic organization, but these technologies still face challenges in terms of throughput, sensitivity, and absolute quantification (Chen et al., 2023).

An even greater challenge lies in the real-time imaging of lipid peroxidation *in vivo*. Ferroptosis is a dynamic process, and our current ability to monitor it relies heavily on static, end-point measurements from biopsies or preclinical models. To truly understand its kinetics and to assess therapeutic efficacy non-invasively, we require technologies that can visualize the key biochemical events of ferroptosis as they happen within a living organism. While significant progress has been made with specific fluorescent probes for microscopy, their translation for deep-tissue or whole-body imaging in patients is largely unfeasible due to poor tissue penetration of light. The development of novel PET and MRI contrast agents that can specifically detect lipid hydroperoxides or other hallmarks of ferroptotic cells remains a holy grail in the field (Paez-Perez et al., 2023). Overcoming these technical barriers is not a minor point of refinement is essential. The ability to spatially resolve the lipid crosstalk within the TME and to dynamically image the process of ferroptosis in response to therapy will be transformative, enabling the rational design of combination strategies, the identification of predictive biomarkers of response, and ultimately, the successful clinical implementation of ferroptosis-based treatments.

### 8.3 New research frontiers

A paradigm shift in our ability to understand the complex network leading the PC-ferroptosis axis has been driven by the application of unbiased, genome-wide CRISPR-based functional genomics screens. These powerful discovery platforms move beyond candidate-gene approaches, allowing for a comprehensive and assumption-free interrogation of the entire genome to identify novel genes and pathways that either sensitize or confer resistance to ferroptotic cell death. By utilizing pooled CRISPR-Cas9 libraries that target every gene for knockout (CRISPRko), transcriptional inhibition (CRISPRi), or activation (CRISPRa), researchers can subject a population of cancer cells to a pro-ferroptotic agent like erastin or RSL3 and subsequently identify which genetic perturbations are enriched or depleted in the surviving cell population via next-generation sequencing. This approach has not only validated the central roles of known players like GPX4 and ACSL4 but has, more importantly, illuminated entirely new and unexpected layers of regulation (Qiu et al., 2024).

The transformative power of this methodology is best exemplified by the landmark screens that led to the independent discovery of the FSP1-CoQ10-NAD(P)H axis, a powerful antioxidant system that operates in parallel to and is entirely independent of the canonical GPX4 pathway. Multiple research groups, employing distinct CRISPR screening strategies, converged on ferroptosis suppressor protein 1 (FSP1, formally known as AIFM2) as a potent ferroptosis resistance factor. Subsequent mechanistic work revealed that FSP1 is a Coenzyme Q10 oxidoreductase that utilizes NAD(P)H to regenerate the reduced form of CoQ10, a lipophilic radical-trapping antioxidant that quenches lipid peroxyl radicals directly within cellular membranes, thereby representing a novel and distinct line of defense (Knott et al., 2018; Doll et al., 2019). These panels have also successfully identified crucial regulators of lipid metabolism, iron homeostasis, and selenocysteine biosynthesis, providing a holistic and integrated map of the ferroptosis regulatory network. Future applications of this technology will undoubtedly involve increasingly sophisticated panel designs, including their use in in vivo models to identify context-dependent regulators within a physiological TME, and in co-culture systems to uncover genes that mediate stromal-driven resistance, ensuring that our map of this intricate cell death modality continues to expand in resolution and complexity.

Moving beyond the discovery of natural regulatory routes to engineer and program this cell death modality with unprecedented precision. The goal is to design therapeutic systems that can selectively induce ferroptosis only in malignant cells, thereby maximizing efficacy while minimizing off-target toxicity. This could involve creating synthetic gene circuits that place key pro-ferroptotic genes, such as an unregulated version of ACSL4 or a dominant-negative GPX4, under the control of a tumor-specific promoter like a promoter that is only active in hypoxic conditions or in the presence of an oncogenic driver. Such a circuit would function as a logic gate, ensuring that the lethal burden is only deployed upon sensing a specific cancer-associated molecular signature (Wang et al., 2023). This concept can be extended to cellular therapies. CAR-T cells could be engineered not only to recognize tumor antigens but also to deliver a synthetic payload such as an enzyme that degrades glutathione, or an exosome filled with a pro-ferroptotic lipid directly into the tumor microenvironment upon target engagement. These smart therapies could be designed to be self-regulating, transforming the challenge of therapy resistance into a new opportunity for targeted cell killing and representing the ultimate convergence of immunology, metabolism, and bioengineering (Dharani et al., 2024).

Bridging the gap between simplistic 2D cell culture and the immense complexity of an *in vivo* tumor requires the development and application of more physiologically relevant preclinical models. The future of studying the PC-ferroptosis axis within the tumor-stroma context lies in the use of sophisticated co-culture techniques and, most powerfully, patient-derived organoid (PDO) systems. Three-dimensional organoids, which can be grown directly from a patient’s tumor biopsy, faithfully recapitulate the genomic landscape, cellular architecture, and differentiation state of the original malignancy (Boj et al., 2015). By co-culturing these PDOs with relevant stromal cell types such as cancer-associated fibroblasts, endothelial cells, and immune cells sourced from the same patient it is possible to build personalized, *in vitro* avatars of a patient’s tumor ecosystem. These advanced models provide an unparalleled platform for investigating the intercellular lipid crosstalk and antioxidant sharing that reinforces ferroptosis resistance in a controlled yet highly relevant setting. They allow researchers to mechanistically dissect how stromal cells modulate the ferroptotic sensitivity of cancer cells and to test the efficacy of combination therapies like ferroptosis inducer coupled with an ICI on a patient-specific basis. As these organoid co-culture models become increasingly sophisticated, incorporating vascularization and stable immune infiltration, they will serve as indispensable tools for both fundamental discovery and for a new era of personalized oncology, enabling the preclinical selection of the most effective therapeutic strategies personalized to the unique metabolic vulnerabilities of an individual’s cancer (Magré et al., 2023).

## 9 Conclusion

The phosphatidylcholine ferroptosis pathway axis functions as a central metabolic checkpoint, closely integrated into cellular regulation, with major implications for sensitivity to ferroptosis and for the interactions between tumors and surrounding supportive cells. At its core, this axis maintains a balance of metabolic pathways that determine a cell vulnerability to ferroptosis, which is a distinct form of programmed cell death driven by iron-dependent lipid damage. By regulating the levels of critical metabolites and enzymes, the phosphatidylcholine ferroptosis pathway axis can either protect cells from this destructive process or make them highly susceptible to it. Beyond its role in controlling cell death, the phosphatidylcholine ferroptosis pathway axis strongly influences the tumor microenvironment, which is a complex and dynamic network that shapes how cancers grow and respond to treatment. Tumor cells constantly communicate with nearby supportive cells such as fibroblasts, immune cells, and blood vessel cells. These interactions can drive tumor growth and spread, but they also create opportunities for therapeutic intervention. The phosphatidylcholine ferroptosis pathway axis plays a central part in this communication, shaping the metabolism of both tumor cells and surrounding supportive cells, and ultimately determining whether the environment favors or hinders cancer progression. Targeting this axis offers a powerful therapeutic opportunity. By shifting the balance toward greater sensitivity to ferroptosis, tumor cells that are normally resistant could become more vulnerable to standard treatments such as chemotherapy or radiation. In addition, by altering the metabolism of supportive cells, the tumor environment itself could be reprogrammed from one that promotes cancer growth to one that suppresses it, thereby limiting progression and spread. However, fully realizing the therapeutic promise of the phosphatidylcholine ferroptosis pathway axis requires a deeper understanding of its diverse roles. A key challenge is to clarify how this axis operates differently in tumor cells, cancer-associated fibroblasts, and immune cells such as T-cells. Defining these specific roles is essential for designing precise interventions that maximize effectiveness while minimizing unintended side effects. Equally important is the identification of concrete intervention points within the axis, such as enzymes, transport proteins, or regulatory molecules that can be targeted with drugs or genetic tools. Mapping these targets will be the critical step in moving from theoretical understanding to clinical application, with the potential to transform cancer treatment.
